# Supplementary material for: Clinicoprognostic and Histopathological Features of Guttate and Plaque Psoriasis Based on PD-1 Expression
Source: J Clin Med. 2021 Nov 7;10(21):5200. doi: 10.3390/jcm10215200 (PMC8584888; doi:10.3390/jcm10215200)
Supplement: Supplementary file 1 [file jcm-10-05200-s001.zip › jcm-1372407-supplementary.pdf]

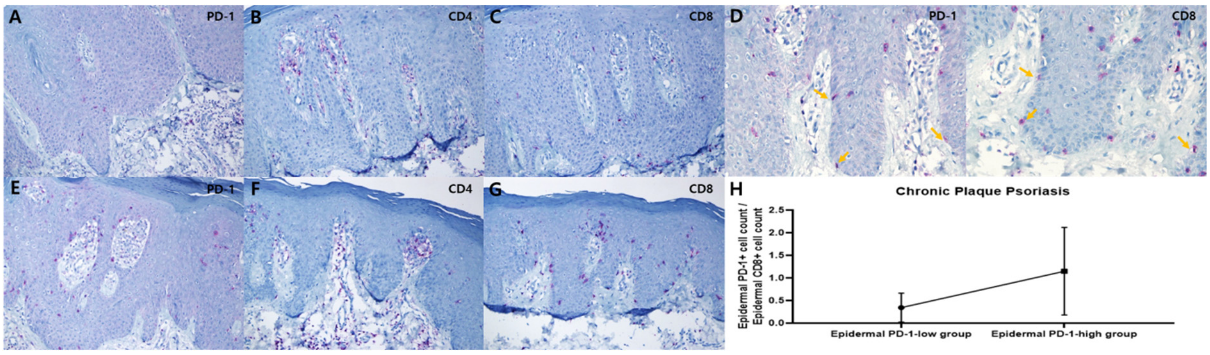

Figure S1. The T cell landscape in representative patients with chronic plaque psoriasis (CPP). **(A)** A patient with CPP of epidermal PD-1-high group showed high epidermal PD-1 expression with some dermal PD-1 expression (magnification X100). **(B)** IHC staining for CD4 was mainly positive for dermal T cells (magnification X100). **(C)** CD8 was mainly positive for epidermal T cells (magnification X100). **(D)** Epidermal PD-1 positive cells seemed to be positive for CD8 in IHC of nearby sections as indicated by arrows (magnification X400). **(E)** Another patient with CPP of epidermal PD-1-high group showed high epidermal PD-1 expression with low dermal PD-1 expression (magnification X100). **(F)** The majority of T cells in dermis were positive for CD4 (magnification X100). **(G)** The majority of T cells in epidermis were positive for CD8 (magnification X100). **(H)** To normalize epidermal PD-1 positive cells, each number of epidermal PD-1 positive cells was devided by the number of epidermal CD8 positive cells. Epidermal high-PD-1 group tended to show higher number of epidermal PD-1 positive cells to number of epidermal CD8 positive cells, compared with epidermal low-PD-1 group.

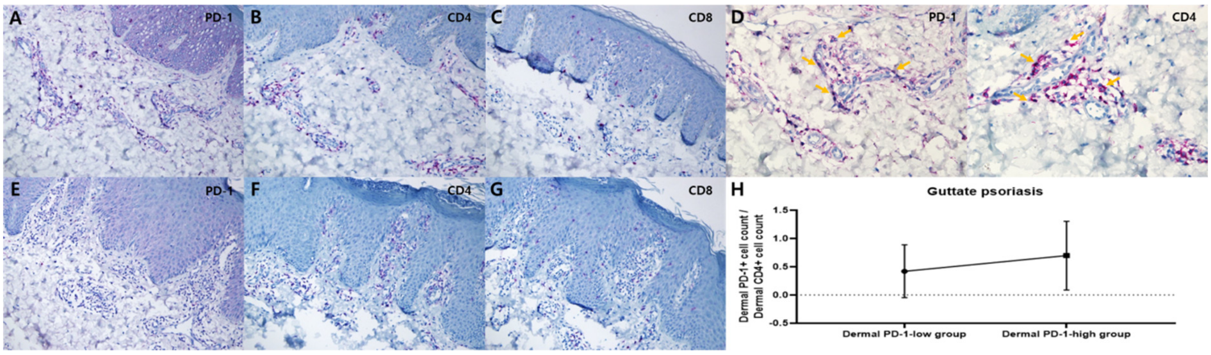

Figure S2. The T cell landscape in representative patients with guttate psoriasis (GP). **(A)** A patient with GP of dermal PD-1-high group showed high dermal PD-1 expression with some epidermal PD-1 expression (magnification X100). **(B)** The majority of T cells in dermis were positive for CD4 (magnification X100). **(C)** The majority of T cells in epidermis were positive for CD8 which are less compared with those of CPP (magnification X100). **(D)** Dermal PD-1 positive cells were also positive for CD4 in IHC of nearby sections as indicated by arrows (magnification X400). **(E)** Another patient with GP of dermal PD-1-high group showed high dermal PD-1 expression with no epidermal PD-1 expression (magnification X 100). **(F)** IHC staining for CD4 was mainly positive for dermal T cells (magnification X100). **(G)** IHC staining for CD8 was mainly positive for epidermal T cells (magnification X100). **(H)** To normalize dermal PD-1 positive cells, each number of dermal PD-1 positive cells was devided by the number of dermal CD4 positive cells. Dermal high-PD-1 group tended to show higher number of dermal PD-1 positive cells to number of dermal CD4 positive cells, compared with dermal low-PD-1 group..

Table S1. Primers used for quantitative real-time polymerase chain reaction analysis.

Primer	Gene name	Gene ID	Forward sequence (5′ to 3′)	Reverse sequence (5′ to 3′)
S100A8	*S100A8*	6279	GGGATGACCTGAAGAAATTGCTA	TGTTGATATCCAACTCTTTGAACCA
PD-L1	*CD274*	29126	TGGCATTTGCTGAACGCATTT	TGCAGCCAGGTCTAATTGTTTT

Abbreviations: PD-1, programmed cell death protein-1; PD-L1, programmed cell death-ligand 1.

Table S2. Clinical characteristics of patients with chronic plaque psoriasis according to the dermal expression level of PD-1.

Characteristics	Dermal PD-1-low group (*n* = 16)	Dermal PD-1-high group (*n* = 13)	*p*-Value
Sex (*n* (%))			
Male	12 (75.0)	9 (69.2)	0.526
Female	4 (25.0)	4 (30.8)	
Age (years)			0.232
Range	12–81	17–66	
Mean ± SD	43.13 ± 18.01	49.54 ± 15.37	
Family history of psoriasis			
Yes	1 (6.3)	0 (0.0)	0.552
No	15 (93.8)	13 (100.0)	
Preceding upper respiratory infection			
Yes	0 (0.0)	1 (7.7)	0.448
No	16 (100.0)	12 (92.3)	
PASI score			0.537
Range	1.2–41.8	5.6–20.8	
Mean ± SD	11.91 ± 10.58	12.03 ± 4.86	
Pruritus			
Yes	15 (93.8)	11 (84.6)	0.420
No	1 (6.3)	2 (15.4)	
Disease duration (months)			0.101
Range	6-360	1-480	
Mean ± SD	118.75 ± 124.89	206.39 ± 140.24	

Abbreviations: PD-1, programmed cell death protein-1; SD, standard deviation; PASI, Psoriasis Area and Severity Index.

Table S3. Histopathological characteristics of patients with chronic plaque psoriasis according to the dermal expression level of PD-1.

Characteristics	Dermal PD-1-low group (*n* = 16)	Dermal PD-1-high group (*n* = 13)	*p*-value
Epidermal thickness (μm)			0.559
Range	138.81–337.170	127.92–475.04	
Mean ± SD	231.72 ± 63.86	264.87 ± 105.38	
Horny layer thickness (μm)			
Range	11.63–117.40	27.16–163.65	0.056
Mean ± SD	42.97 ± 28.67	62.36 ± 36.47	
Rete ridge count (n)			
Range	8–15	8–15	0.351
Mean ± SD	11.94 ± 2.08	11.69 ± 1.70	
Cellular infiltration grading			
Range	1–3	1-3	0.042*
Mean ± SD	1.63 ± 0.72	2.15 ± 0.55	
Vessel dilatation grading			
Range	1–2	2–3	0.129
Mean ± SD	1.62 ± 0.50	2.00 ± 0.41	

* Statistically significant. Abbreviations: PD-1, programmed cell death protein-1; SD, standard deviation.
